# Supplementary material for: Deletion of wfs1 Impairs Oligodendrocyte Precursor Cells Dorsal Distribution and Myelination Through the wfs1-hmgcs1 Axis in Zebrafish
Source: Biology (Basel). 2026 Aug 16;15(16):1402. doi: 10.3390/biology15161402 (PMC13510151; doi:10.3390/biology15161402)
Supplement: Supplementary file 1 [file biology-15-01402-s001.zip › File S1-Transcriptome sequencing data and differentially expressed genes (DEGs) in wfs1-- zebrafish at 5 dpf/2.KEGG/wfs1KOvsWT/GSEA/wfs1KO/gsea_report_for_wfs1KO_1700447271053.html]

Report for wfs1KO 1700447271053 [GSEA]


| GS   follow link to MSigDB | GS DETAILS | SIZE | ES | NES | NOM p-val | FDR q-val | FWER p-val | RANK AT MAX | LEADING EDGE || 1 | RNA\_DEGRADATION(DRE03018) | Details ... | 108 | 0.40 | 1.96 | 0.000 | 0.028 | 0.010 | 1722 | tags=14%, list=5%, signal=15% |
| 2 | PHOSPHATIDYLINOSITOL\_SIGNALING\_SYSTEM(DRE04070) | Details ... | 157 | 0.34 | 1.90 | 0.000 | 0.064 | 0.079 | 2115 | tags=10%, list=7%, signal=11% |
| 3 | SNARE\_INTERACTIONS\_IN\_VESICULAR\_TRANSPORT(DRE04130) | Details ... | 56 | 0.50 | 1.87 | 0.000 | 0.056 | 0.089 | 2513 | tags=21%, list=8%, signal=23% |
| 4 | STEROID\_BIOSYNTHESIS(DRE00100) | Details ... | 24 | 0.57 | 1.74 | 0.000 | 0.101 | 0.170 | 3921 | tags=38%, list=12%, signal=43% |
| 5 | CARBON\_METABOLISM(DRE01200) | Details ... | 173 | 0.35 | 1.71 | 0.000 | 0.093 | 0.191 | 4480 | tags=22%, list=14%, signal=25% |
| 6 | CYSTEINE\_AND\_METHIONINE\_METABOLISM(DRE00270) | Details ... | 73 | 0.36 | 1.70 | 0.000 | 0.084 | 0.191 | 1121 | tags=14%, list=4%, signal=14% |
| 7 | GLUTATHIONE\_METABOLISM(DRE00480) | Details ... | 85 | 0.41 | 1.68 | 0.000 | 0.095 | 0.224 | 3023 | tags=16%, list=9%, signal=18% |
| 8 | TERPENOID\_BACKBONE\_BIOSYNTHESIS(DRE00900) | Details ... | 26 | 0.43 | 1.68 | 0.026 | 0.087 | 0.243 | 1388 | tags=15%, list=4%, signal=16% |
| 9 | INTESTINAL\_IMMUNE\_NETWORK\_FOR\_IGA\_PRODUCTION(DRE04672) | Details ... | 37 | 0.53 | 1.66 | 0.000 | 0.090 | 0.295 | 2055 | tags=27%, list=6%, signal=29% |
| 10 | CITRATE\_CYCLE\_(TCA\_CYCLE)(DRE00020) | Details ... | 47 | 0.40 | 1.63 | 0.000 | 0.101 | 0.325 | 3094 | tags=23%, list=10%, signal=26% |
| 11 | NUCLEOCYTOPLASMIC\_TRANSPORT(DRE03013) | Details ... | 129 | 0.28 | 1.61 | 0.000 | 0.114 | 0.356 | 2292 | tags=9%, list=7%, signal=9% |
| 12 | FATTY\_ACID\_METABOLISM(DRE01212) | Details ... | 91 | 0.35 | 1.51 | 0.036 | 0.237 | 0.603 | 5108 | tags=27%, list=16%, signal=33% |
| 13 | GLYOXYLATE\_AND\_DICARBOXYLATE\_METABOLISM(DRE00630) | Details ... | 46 | 0.39 | 1.51 | 0.068 | 0.223 | 0.618 | 4462 | tags=22%, list=14%, signal=25% |
| 14 | SALMONELLA\_INFECTION(DRE05132) | Details ... | 406 | 0.24 | 1.50 | 0.000 | 0.220 | 0.637 | 3362 | tags=10%, list=11%, signal=11% |
| 15 | PROPANOATE\_METABOLISM(DRE00640) | Details ... | 41 | 0.45 | 1.50 | 0.000 | 0.210 | 0.637 | 6280 | tags=32%, list=20%, signal=39% |
| 16 | PYRUVATE\_METABOLISM(DRE00620) | Details ... | 68 | 0.40 | 1.50 | 0.031 | 0.200 | 0.652 | 4243 | tags=24%, list=13%, signal=27% |
| 17 | VALINE\_\_LEUCINE\_AND\_ISOLEUCINE\_DEGRADATION(DRE00280) | Details ... | 71 | 0.33 | 1.49 | 0.016 | 0.202 | 0.689 | 5134 | tags=25%, list=16%, signal=30% |
| 18 | PENTOSE\_PHOSPHATE\_PATHWAY(DRE00030) | Details ... | 45 | 0.36 | 1.49 | 0.044 | 0.196 | 0.689 | 730 | tags=11%, list=2%, signal=11% |
| 19 | STARCH\_AND\_SUCROSE\_METABOLISM(DRE00500) | Details ... | 44 | 0.43 | 1.49 | 0.032 | 0.187 | 0.689 | 5390 | tags=32%, list=17%, signal=38% |
| 20 | GLYCEROPHOSPHOLIPID\_METABOLISM(DRE00564) | Details ... | 135 | 0.32 | 1.48 | 0.000 | 0.184 | 0.711 | 6579 | tags=27%, list=21%, signal=33% |
| 21 | MRNA\_SURVEILLANCE\_PATHWAY(DRE03015) | Details ... | 118 | 0.29 | 1.48 | 0.000 | 0.184 | 0.729 | 1654 | tags=8%, list=5%, signal=8% |
| 22 | PROTEIN\_PROCESSING\_IN\_ENDOPLASMIC\_RETICULUM(DRE04141) | Details ... | 241 | 0.25 | 1.48 | 0.000 | 0.176 | 0.729 | 5229 | tags=16%, list=16%, signal=19% |
| 23 | P53\_SIGNALING\_PATHWAY(DRE04115) | Details ... | 101 | 0.30 | 1.47 | 0.000 | 0.172 | 0.729 | 3458 | tags=16%, list=11%, signal=18% |
| 24 | GLYCOSAMINOGLYCAN\_BIOSYNTHESIS\_\_\_CHONDROITIN\_SULFATE\_\_\_DERMATAN\_SULFATE(DRE00532) | Details ... | 31 | 0.46 | 1.47 | 0.082 | 0.169 | 0.769 | 7006 | tags=35%, list=22%, signal=45% |
| 25 | BUTANOATE\_METABOLISM(DRE00650) | Details ... | 26 | 0.54 | 1.46 | 0.111 | 0.176 | 0.807 | 4565 | tags=27%, list=14%, signal=31% |
| 26 | TYROSINE\_METABOLISM(DRE00350) | Details ... | 50 | 0.37 | 1.45 | 0.073 | 0.174 | 0.807 | 3009 | tags=20%, list=9%, signal=22% |
| 27 | RIBOSOME\_BIOGENESIS\_IN\_EUKARYOTES(DRE03008) | Details ... | 83 | 0.28 | 1.43 | 0.036 | 0.189 | 0.829 | 896 | tags=5%, list=3%, signal=5% |
| 28 | GLYCOSPHINGOLIPID\_BIOSYNTHESIS\_\_\_GLOBO\_AND\_ISOGLOBO\_SERIES(DRE00603) | Details ... | 20 | 0.49 | 1.41 | 0.110 | 0.210 | 0.858 | 1072 | tags=15%, list=3%, signal=16% |
| 29 | ABC\_TRANSPORTERS(DRE02010) | Details ... | 63 | 0.41 | 1.41 | 0.059 | 0.210 | 0.875 | 4759 | tags=29%, list=15%, signal=34% |
| 30 | NUCLEOTIDE\_METABOLISM(DRE01232) | Details ... | 131 | 0.31 | 1.39 | 0.026 | 0.232 | 0.906 | 3561 | tags=15%, list=11%, signal=17% |
| 31 | GLYCEROLIPID\_METABOLISM(DRE00561) | Details ... | 89 | 0.32 | 1.37 | 0.015 | 0.247 | 0.906 | 5326 | tags=24%, list=17%, signal=28% |
| 32 | INOSITOL\_PHOSPHATE\_METABOLISM(DRE00562) | Details ... | 123 | 0.29 | 1.36 | 0.045 | 0.250 | 0.922 | 2115 | tags=8%, list=7%, signal=9% |
| 33 | CELL\_CYCLE(DRE04110) | Details ... | 229 | 0.23 | 1.35 | 0.000 | 0.259 | 0.931 | 4099 | tags=13%, list=13%, signal=15% |
| 34 | TRYPTOPHAN\_METABOLISM(DRE00380) | Details ... | 67 | 0.31 | 1.34 | 0.087 | 0.269 | 0.931 | 6016 | tags=37%, list=19%, signal=46% |
| 35 | GLYCOSAMINOGLYCAN\_BIOSYNTHESIS\_\_\_KERATAN\_SULFATE(DRE00533) | Details ... | 23 | 0.38 | 1.34 | 0.035 | 0.261 | 0.931 | 5115 | tags=26%, list=16%, signal=31% |
| 36 | FATTY\_ACID\_ELONGATION(DRE00062) | Details ... | 46 | 0.38 | 1.34 | 0.157 | 0.258 | 0.931 | 5292 | tags=30%, list=17%, signal=36% |
| 37 | METABOLIC\_PATHWAYS(DRE01100) | Details ... | 2186 | 0.25 | 1.34 | 0.000 | 0.253 | 0.931 | 5195 | tags=18%, list=16%, signal=20% |
| 38 | FOLATE\_BIOSYNTHESIS(DRE00790) | Details ... | 38 | 0.34 | 1.33 | 0.157 | 0.264 | 0.946 | 1560 | tags=13%, list=5%, signal=14% |
| 39 | FANCONI\_ANEMIA\_PATHWAY(DRE03460) | Details ... | 63 | 0.36 | 1.32 | 0.028 | 0.271 | 0.946 | 2491 | tags=14%, list=8%, signal=15% |
| 40 | PPAR\_SIGNALING\_PATHWAY(DRE03320) | Details ... | 106 | 0.26 | 1.31 | 0.085 | 0.283 | 0.957 | 3698 | tags=16%, list=12%, signal=18% |
| 41 | FATTY\_ACID\_BIOSYNTHESIS(DRE00061) | Details ... | 28 | 0.34 | 1.30 | 0.033 | 0.286 | 0.957 | 5835 | tags=25%, list=18%, signal=31% |
| 42 | NON\_HOMOLOGOUS\_END\_JOINING(DRE03450) | Details ... | 16 | 0.48 | 1.29 | 0.091 | 0.297 | 0.981 | 5630 | tags=31%, list=18%, signal=38% |
| 43 | PHENYLALANINE\_METABOLISM(DRE00360) | Details ... | 22 | 0.43 | 1.29 | 0.182 | 0.294 | 0.981 | 2167 | tags=23%, list=7%, signal=24% |
| 44 | GLYCINE\_\_SERINE\_AND\_THREONINE\_METABOLISM(DRE00260) | Details ... | 56 | 0.30 | 1.27 | 0.082 | 0.308 | 0.981 | 2618 | tags=13%, list=8%, signal=14% |
| 45 | TAURINE\_AND\_HYPOTAURINE\_METABOLISM(DRE00430) | Details ... | 23 | 0.47 | 1.26 | 0.163 | 0.316 | 0.981 | 10290 | tags=52%, list=32%, signal=77% |
| 46 | NOTCH\_SIGNALING\_PATHWAY(DRE04330) | Details ... | 93 | 0.32 | 1.25 | 0.168 | 0.326 | 0.981 | 3778 | tags=15%, list=12%, signal=17% |
| 47 | ARACHIDONIC\_ACID\_METABOLISM(DRE00590) | Details ... | 72 | 0.34 | 1.25 | 0.110 | 0.321 | 0.981 | 2612 | tags=19%, list=8%, signal=21% |
| 48 | SPLICEOSOME(DRE03040) | Details ... | 164 | 0.22 | 1.24 | 0.108 | 0.326 | 0.981 | 2952 | tags=7%, list=9%, signal=7% |
| 49 | HOMOLOGOUS\_RECOMBINATION(DRE03440) | Details ... | 46 | 0.37 | 1.24 | 0.183 | 0.327 | 1.000 | 2429 | tags=17%, list=8%, signal=19% |
| 50 | FOXO\_SIGNALING\_PATHWAY(DRE04068) | Details ... | 210 | 0.24 | 1.24 | 0.154 | 0.321 | 1.000 | 4819 | tags=15%, list=15%, signal=18% |
| 51 | CYTOKINE\_CYTOKINE\_RECEPTOR\_INTERACTION(DRE04060) |  | 242 | 0.30 | 1.23 | 0.016 | 0.333 | 1.000 | 5299 | tags=19%, list=17%, signal=22% |
| 52 | LYSOSOME(DRE04142) |  | 214 | 0.27 | 1.22 | 0.139 | 0.344 | 1.000 | 5549 | tags=21%, list=17%, signal=25% |
| 53 | PURINE\_METABOLISM(DRE00230) |  | 192 | 0.26 | 1.22 | 0.195 | 0.345 | 1.000 | 7184 | tags=22%, list=23%, signal=28% |
| 54 | PROTEASOME(DRE03050) |  | 65 | 0.32 | 1.21 | 0.223 | 0.342 | 1.000 | 1494 | tags=6%, list=5%, signal=6% |
| 55 | ECM\_RECEPTOR\_INTERACTION(DRE04512) |  | 128 | 0.29 | 1.21 | 0.235 | 0.336 | 1.000 | 2305 | tags=9%, list=7%, signal=10% |
| 56 | C\_TYPE\_LECTIN\_RECEPTOR\_SIGNALING\_PATHWAY(DRE04625) |  | 156 | 0.24 | 1.21 | 0.065 | 0.332 | 1.000 | 7293 | tags=27%, list=23%, signal=35% |
| 57 | PEROXISOME(DRE04146) |  | 114 | 0.26 | 1.20 | 0.209 | 0.352 | 1.000 | 7216 | tags=30%, list=23%, signal=38% |
| 58 | PROGESTERONE\_MEDIATED\_OOCYTE\_MATURATION(DRE04914) |  | 133 | 0.24 | 1.20 | 0.154 | 0.347 | 1.000 | 7195 | tags=22%, list=23%, signal=28% |
| 59 | ONE\_CARBON\_POOL\_BY\_FOLATE(DRE00670) |  | 25 | 0.32 | 1.19 | 0.199 | 0.343 | 1.000 | 1969 | tags=12%, list=6%, signal=13% |
| 60 | ETHER\_LIPID\_METABOLISM(DRE00565) |  | 67 | 0.29 | 1.19 | 0.232 | 0.341 | 1.000 | 5502 | tags=24%, list=17%, signal=29% |
| 61 | GLYCOSAMINOGLYCAN\_BIOSYNTHESIS\_\_\_HEPARAN\_SULFATE\_\_\_HEPARIN(DRE00534) |  | 38 | 0.32 | 1.19 | 0.217 | 0.346 | 1.000 | 4567 | tags=21%, list=14%, signal=25% |
| 62 | GNRH\_SIGNALING\_PATHWAY(DRE04912) |  | 158 | 0.22 | 1.18 | 0.092 | 0.351 | 1.000 | 8283 | tags=27%, list=26%, signal=37% |
| 63 | PYRIMIDINE\_METABOLISM(DRE00240) |  | 84 | 0.28 | 1.18 | 0.137 | 0.347 | 1.000 | 3561 | tags=15%, list=11%, signal=17% |
| 64 | ADRENERGIC\_SIGNALING\_IN\_CARDIOMYOCYTES(DRE04261) |  | 301 | 0.22 | 1.17 | 0.124 | 0.354 | 1.000 | 4082 | tags=13%, list=13%, signal=15% |
| 65 | CELLULAR\_SENESCENCE(DRE04218) |  | 234 | 0.21 | 1.17 | 0.066 | 0.351 | 1.000 | 4903 | tags=15%, list=15%, signal=18% |
| 66 | FOCAL\_ADHESION(DRE04510) |  | 354 | 0.22 | 1.17 | 0.123 | 0.351 | 1.000 | 3069 | tags=9%, list=10%, signal=10% |
| 67 | BIOSYNTHESIS\_OF\_UNSATURATED\_FATTY\_ACIDS(DRE01040) |  | 39 | 0.34 | 1.16 | 0.333 | 0.347 | 1.000 | 5292 | tags=28%, list=17%, signal=34% |
| 68 | FATTY\_ACID\_DEGRADATION(DRE00071) |  | 65 | 0.26 | 1.16 | 0.271 | 0.345 | 1.000 | 5134 | tags=22%, list=16%, signal=26% |
| 69 | ADHERENS\_JUNCTION(DRE04520) |  | 194 | 0.23 | 1.14 | 0.265 | 0.381 | 1.000 | 2486 | tags=8%, list=8%, signal=9% |
| 70 | BIOSYNTHESIS\_OF\_COFACTORS(DRE01240) |  | 234 | 0.27 | 1.13 | 0.330 | 0.387 | 1.000 | 3526 | tags=15%, list=11%, signal=16% |
| 71 | MAPK\_SIGNALING\_PATHWAY(DRE04010) |  | 503 | 0.23 | 1.12 | 0.179 | 0.404 | 1.000 | 3939 | tags=14%, list=12%, signal=15% |
| 72 | RIG\_I\_LIKE\_RECEPTOR\_SIGNALING\_PATHWAY(DRE04622) |  | 76 | 0.27 | 1.12 | 0.286 | 0.401 | 1.000 | 5568 | tags=20%, list=17%, signal=24% |
| 73 | MELANOGENESIS(DRE04916) |  | 171 | 0.21 | 1.10 | 0.198 | 0.419 | 1.000 | 3185 | tags=11%, list=10%, signal=12% |
| 74 | ALPHA\_LINOLENIC\_ACID\_METABOLISM(DRE00592) |  | 26 | 0.35 | 1.10 | 0.386 | 0.418 | 1.000 | 5502 | tags=35%, list=17%, signal=42% |
| 75 | VASCULAR\_SMOOTH\_MUSCLE\_CONTRACTION(DRE04270) |  | 226 | 0.22 | 1.09 | 0.191 | 0.427 | 1.000 | 5008 | tags=15%, list=16%, signal=17% |
| 76 | ADIPOCYTOKINE\_SIGNALING\_PATHWAY(DRE04920) |  | 108 | 0.25 | 1.07 | 0.389 | 0.451 | 1.000 | 7033 | tags=25%, list=22%, signal=32% |
| 77 | BIOSYNTHESIS\_OF\_NUCLEOTIDE\_SUGARS(DRE01250) |  | 51 | 0.26 | 1.07 | 0.368 | 0.452 | 1.000 | 4685 | tags=25%, list=15%, signal=30% |
| 78 | AUTOPHAGY\_\_\_ANIMAL(DRE04140) |  | 241 | 0.21 | 1.06 | 0.297 | 0.461 | 1.000 | 5344 | tags=17%, list=17%, signal=20% |
| 79 | MUCIN\_TYPE\_O\_GLYCAN\_BIOSYNTHESIS(DRE00512) |  | 43 | 0.27 | 1.06 | 0.428 | 0.471 | 1.000 | 4969 | tags=21%, list=16%, signal=25% |
| 80 | LINOLEIC\_ACID\_METABOLISM(DRE00591) |  | 33 | 0.37 | 1.05 | 0.380 | 0.470 | 1.000 | 2559 | tags=21%, list=8%, signal=23% |
| 81 | ARGININE\_AND\_PROLINE\_METABOLISM(DRE00330) |  | 81 | 0.24 | 1.03 | 0.456 | 0.509 | 1.000 | 6229 | tags=25%, list=20%, signal=31% |
| 82 | INSULIN\_SIGNALING\_PATHWAY(DRE04910) |  | 212 | 0.21 | 1.03 | 0.382 | 0.504 | 1.000 | 5108 | tags=16%, list=16%, signal=18% |
| 83 | GALACTOSE\_METABOLISM(DRE00052) |  | 43 | 0.30 | 1.03 | 0.389 | 0.501 | 1.000 | 5195 | tags=33%, list=16%, signal=39% |
| 84 | PANTOTHENATE\_AND\_COA\_BIOSYNTHESIS(DRE00770) |  | 28 | 0.30 | 1.02 | 0.381 | 0.501 | 1.000 | 3351 | tags=21%, list=11%, signal=24% |
| 85 | OTHER\_TYPES\_OF\_O\_GLYCAN\_BIOSYNTHESIS(DRE00514) |  | 65 | 0.24 | 1.01 | 0.466 | 0.520 | 1.000 | 5115 | tags=18%, list=16%, signal=22% |
| 86 | AMINO\_SUGAR\_AND\_NUCLEOTIDE\_SUGAR\_METABOLISM(DRE00520) |  | 74 | 0.27 | 1.00 | 0.439 | 0.544 | 1.000 | 7570 | tags=32%, list=24%, signal=42% |
| 87 | CYTOSOLIC\_DNA\_SENSING\_PATHWAY(DRE04623) |  | 72 | 0.25 | 1.00 | 0.334 | 0.540 | 1.000 | 5568 | tags=19%, list=17%, signal=24% |
| 88 | THIAMINE\_METABOLISM(DRE00730) |  | 18 | 0.33 | 0.98 | 0.509 | 0.565 | 1.000 | 1560 | tags=11%, list=5%, signal=12% |
| 89 | PHAGOSOME(DRE04145) |  | 209 | 0.21 | 0.97 | 0.501 | 0.572 | 1.000 | 4934 | tags=15%, list=15%, signal=17% |
| 90 | PHOTOTRANSDUCTION(DRE04744) |  | 57 | 0.31 | 0.96 | 0.427 | 0.589 | 1.000 | 5129 | tags=21%, list=16%, signal=25% |
| 91 | CELL\_ADHESION\_MOLECULES(DRE04514) |  | 204 | 0.20 | 0.93 | 0.594 | 0.636 | 1.000 | 5644 | tags=17%, list=18%, signal=20% |
| 92 | OOCYTE\_MEIOSIS(DRE04114) |  | 182 | 0.18 | 0.92 | 0.686 | 0.645 | 1.000 | 6607 | tags=19%, list=21%, signal=23% |
| 93 | DRUG\_METABOLISM\_\_\_OTHER\_ENZYMES(DRE00983) |  | 124 | 0.26 | 0.91 | 0.496 | 0.663 | 1.000 | 3561 | tags=18%, list=11%, signal=20% |
| 94 | STEROID\_HORMONE\_BIOSYNTHESIS(DRE00140) |  | 91 | 0.29 | 0.91 | 0.509 | 0.660 | 1.000 | 6151 | tags=31%, list=19%, signal=38% |
| 95 | APELIN\_SIGNALING\_PATHWAY(DRE04371) |  | 226 | 0.18 | 0.90 | 0.661 | 0.661 | 1.000 | 3581 | tags=10%, list=11%, signal=11% |
| 96 | RETINOL\_METABOLISM(DRE00830) |  | 104 | 0.28 | 0.87 | 0.592 | 0.726 | 1.000 | 3629 | tags=18%, list=11%, signal=21% |
| 97 | NITROGEN\_METABOLISM(DRE00910) |  | 24 | 0.30 | 0.81 | 0.679 | 0.830 | 1.000 | 1805 | tags=13%, list=6%, signal=13% |
| 98 | PORPHYRIN\_METABOLISM(DRE00860) |  | 74 | 0.24 | 0.79 | 0.587 | 0.856 | 1.000 | 4639 | tags=22%, list=15%, signal=25% |
| 99 | DRUG\_METABOLISM\_\_\_CYTOCHROME\_P450(DRE00982) |  | 85 | 0.26 | 0.79 | 0.730 | 0.849 | 1.000 | 5989 | tags=25%, list=19%, signal=30% |
| 100 | VARIOUS\_TYPES\_OF\_N\_GLYCAN\_BIOSYNTHESIS(DRE00513) |  | 58 | 0.17 | 0.78 | 0.814 | 0.854 | 1.000 | 7623 | tags=19%, list=24%, signal=25% |
| 101 | NICOTINATE\_AND\_NICOTINAMIDE\_METABOLISM(DRE00760) |  | 52 | 0.20 | 0.77 | 0.835 | 0.855 | 1.000 | 5668 | tags=15%, list=18%, signal=19% |
| 102 | TGF\_BETA\_SIGNALING\_PATHWAY(DRE04350) |  | 161 | 0.18 | 0.77 | 0.688 | 0.854 | 1.000 | 5344 | tags=13%, list=17%, signal=16% |
| 103 | PENTOSE\_AND\_GLUCURONATE\_INTERCONVERSIONS(DRE00040) |  | 62 | 0.27 | 0.77 | 0.655 | 0.846 | 1.000 | 3526 | tags=21%, list=11%, signal=24% |
| 104 | METABOLISM\_OF\_XENOBIOTICS\_BY\_CYTOCHROME\_P450(DRE00980) |  | 85 | 0.24 | 0.75 | 0.668 | 0.851 | 1.000 | 3526 | tags=16%, list=11%, signal=18% |
Table: Gene sets enriched in phenotype
 **wfs1KO (4 samples)
 **[plain text format]****
